# Fibre-Optic Surface Plasmon Resonance Biosensor for Monoclonal Antibody Titer Quantification

**DOI:** 10.3390/bios11100383

**Published:** 2021-10-10

**Authors:** Thai Thao Ly, Yinlan Ruan, Bobo Du, Peipei Jia, Hu Zhang

**Affiliations:** 1 School of Chemical Engineering and Advanced Materials, University of Adelaide, Adelaide 5005, Australia; thaithao.ly@adelaide.edu.au; 2 Institute for Photonics and Advanced Sensing, University of Adelaide, Adelaide 5005, Australia; 3 School of Electronic Engineering and Automation, Guilin University of Electronic University, Guilin 541004, China; 4 Key Laboratory for Physical Electronics and Devices of the Ministry of Education, School of Electronic Science and Engineering, Xi’an Jiaotong University, Xi’an 710049, China; bobo.du@xjtu.edu.cn; 5 Shenzhen Institute for Advanced Study, University of Electronic Science and Technology of China, Shenzhen 518000, China; jiapeipei@uestc.edu.cn; 6 Keck Graduate Institute, Claremont, CA 91711, USA

**Keywords:** antibody titer, optical fibre, SPR biosensor, process analytical technology

## Abstract

An extraordinary optical transmission fibre-optic surface plasmon resonance biosensing platform was engineered to improve its portability and sensitivity, and was applied to monitor the concentrations of monoclonal antibodies (Mabs). By refining the fabricating procedure and changing the material of the flow cell and the components of the optical fibre, the biosensor is portable and robust to external interference. After the implementation of an effective template cleaning procedure and precise control during the fabrication process, a consistent sensitivity of 509 ± 5 nm per refractive index unit (nm/RIU) was achieved. The biosensor can detect the Mab with a limit of detection (LOD) of 0.44 µg/mL. The results show that the biosensor is a potential tool for the rapid quantification of Mab titers. The biosensor can be regenerated at least 10 times with 10 mM glycine (pH = 2.5), and consistent signal changes were obtained after regeneration. Moreover, the employment of a spacer arm SM(PEG)2, used for immobilising protein A onto the gold film, was demonstrated to be unable to improve the detecting sensitivity; thus, a simple procedure without the spacer arm could be used to prepare the protein A-based biosensor. Our results demonstrate that the fibre-optic surface plasmon resonance biosensor is competent for the real-time and on-line monitoring of antibody titers in the future as a process analytical technologies (PATs) tool for bioprocess developments and the manufacture of therapeutic antibodies.

## 1. Introduction

The growth in continuous processes for therapeutic Mab production demands innovative/disruptive technologies to monitor the Mab concentration. However, there are few reliable PATs that can be used to measure the Mab titer. The rapid at/in/on-line determination of a Mab titer is important because it provides real-time information to monitor the product yield for immediate decision-making during production [1]. In addition, it reflects the impacts of critical/key process parameters (CPPs/KPPs) on the process efficiency. Ideal titer measurement methods should provide Mab titers in a timely manner, with acceptable measuring frequency, accuracy, and precision. Moreover, they need to have sufficient reproducibility and comply with the Good Manufacturing Practices (GMP) requirements and pharmaceutical regulations. An appropriate in/at/on-line titer method should present low-to-no risk of contaminating the process in a GMP production environment [2]. Portability and ease of maintenance are also crucial factors that influence the choice of titer detection method. The production site usually has limited space; therefore, only compact analytical equipment can be coupled with bioreactors or downstream equipment in/at/on-line. Moreover, in case of failure, the instrument should be able to be repaired or replaced quickly to avoid delays [1]. Many available Mab titer measurement methods such as Patrol ultraperformance liquid chromatography and Idex Tridex protein analyser satisfy some of these crucial factors; however, they have distinct drawbacks such as high capital and maintenance costs, a substantially spacious dimension, poor accuracy, and low reliability [1].

Fibre-optic surface plasmon resonance (SPR) biosensors could emerge as a potential PATs tool to measure Mabs titers. They can rapidly and selectively measure the Mab concentrations through the binding of specific biological factors with outstanding robustness and decent sensitivity. Furthermore, fibre-optic SPR biosensors are very portable and could readily be implemented on-line in a GMP environment. The small size of the biosensors means that a backup biosensor can be accommodated on-site in case of instrumental failure. 

SPR biosensors employ a metal film as their sensing surface. The metal surface has free electrons that form collective oscillations when excited by an incident light source. Collective oscillations of free electrons are also known as SPR, a principal mechanism behind many optical sensors [3]. The excitation of electron oscillations at a metal surface creates an electromagnetic field that is highly sensitive to a subtle change in the refractive index at the metal–dielectric interface [3]. Therefore, SPR biosensors have excellent sensitivity.

SPR has been well-developed and commercialised for products such as Biacore by Cytiva Life Sciences. Biacore is used to analyse the interaction between biomolecules with excellent sensitivity. However, it is based on a continuous gold film and requires the incident optical beam to illuminate the sample at a specific angle relative to the film to excite the SPR, which leads to a complicated optical setup. Additionally, it is only applicable in a laboratory environment because of its bulky size and high cost. Extraordinary optical transmission (EOT) biosensors are a family member of SPR biosensors, which were developed based on an optical phenomenon observed by Ebbesen, et al. [4]. EOT occurs when light incidents a metal film with a sub-wavelength nanohole array at a normal angle [4] or a bevelled angle [5]. Compared to the SPR biosensors based on the continuous gold film, EOT biosensors based on localised SPR do not require a bulky and fragile prism, and their SPR modes are excited by the beam illuminating the nanohole film with a very flexible angle. This makes them much simpler than SPR biosensors. Furthermore, the nanohole array provides a larger surface area for binding the analytes and facilitates the detection of analytes at a more comprehensive detection area [6]. Consequently, EOT biosensors have attracted interest as a portable alternative to conventional SPR biosensors. 

The development of EOT biosensors becomes more attractive as the technique for surface fabrication with nanohole arrays evolves. A gold film with nanohole array for EOT sensors is usually fabricated by a focused ion beam (FIB) [7,8,9,10,11,12,13,14,15,16,17,18,19] or lithography [20,21,22,23,24,25,26,27]. These methods are very costly and time-consuming [27,28], as they require a dedicated instrument to fabricate the surface. Moreover, they cannot fabricate long-order patterns with good precision [28] and the sensing surface usually has considerable roughness, which may impact the sensing sensitivity [29]. In recent years, template-stripping (also known as template transfer) has been extensively as an alternative method for fabricating the gold film with nanohole arrays to gradually replace the FIB method or lithography [5,28,30,31,32,33,34,35,36]. Template-stripping was first introduced by Hegner, et al. [37] to generate an ultra-flat gold surface. Nagpal, Lindquist, Oh and Norris [29] employed this technique to create an ultra-smooth metal surface with patterns. This method is cost effective since the silicon template can be reused multiple times. It also allows for mass production of the metal film with a smooth surface [28,29,31]. 

The fibre-optic SPR-EOT technique is derived from recently established EOT biosensors and conventional SPR biosensors [3]. In most EOT-based biosensors, a nanopatterned gold film is fabricated onto a glass slide, and a polydimethylsiloxane (PDMS) microfluidic chip is attached to the glass slide to form a microfluidic chip. The microfluidic chip could have multiple channels to simultaneously measure many samples [30,35]. However, such a setup with microfluidic chips is not adequately portable. The microfluidic chip must be fixed to align with the focused region of a microscope and the imaging sensor. The biosensor becomes portable, cheap, and robust through the implementation of a gold film on the tip of optical fibres instead of glass slides. Fibre-optic SPR-EOT biosensors have been developed and applied in biomolecular detection by several research groups [7,9,15,18,19,34]. Herein, template-stripping was used to fabricate a smooth gold-sensing surface with a nanohole array on the tip of an optical fibre for our fibre-optic SOR-EOT biosensors. Our EOT biosensing platform was derived from the platform developed by Jia and Yang [33]. 

Our subsequent works have demonstrated that this platform is robust and reliable for practical measurements of large biomolecules. We first improved the reliability and portability of the fibre-optic SPR-EOT biosensor, minimised the external interference via design modifications of the biosensing platform, and elaborated its potential as a PAT tool for detecting Mab titers in a bioreactor. Engineering modifications were made to improve the stability of the biosensor during operation, thus enhancing the overall reproducibility and reliability. After obtaining a biosensing platform with stable operating conditions and excellent reproducibility, the biosensor was applied to detect Mabs at a wide range of concentrations (10^−5^ to 10^−2^ mg/mL). A consistent detection limit of 0.44 µg/mL across three individual sensing probes confirmed the reliability and reproducibility of the biosensor. The results show that our fibre-optic SPR-EOT biosensor can detect a Mab with decent reproducibility and has excellent potential to become a portable at-line PAT device.

## 2. Materials and Methods

### 2.1. Fabricating the Gold Sensing Surface

The template was a nanopatterned silicon stamp from Lightsmyth (S2D-18D3-0808-350-P, Eugene, OR, USA) with a hexagonal lattice with a period of 700 nm and a hole diameter of 200 nm. The templates were first cleaned in a Piranha solution for 30 min to remove the residual of the epoxy glue, then in an Aqua Regia solution for 30 min to remove the residual of gold, and finally in a Piranha solution for 30 min to remove organic contaminants on the template surface. All cleaning steps were carried out in a sonicating bath to maximise the cleaning efficiency. The template quality after each cleaning step was examined under an optical microscope.

After cleaning, the gold film with 100 nm thickness was deposited on the template using a thermal evaporator (Quorum Technology-K975X Turbo-Pumped Thermal Evaporator, Laughton, UK). The gold nanostructure on the template was then transferred onto the end facet of an optical fibre using epoxy glue. 

### 2.2. Fabricating and Assembling the Flow Cell

A chamber of the flow cell designed with dimensions of 10 × 6 × 6 mm was printed from a photopolymer resin (Formlabs, Somerville, MA, USA) by a 3D printer (Form 2, Formlabs, Somerville, MA, USA), as shown in Figure S1 (Supplementary document). Each complete flow cell consisted of one optical fibre attached with a sensing gold film as the input end, one bare optical fibre to collect the transmission signal from the gold film on the input fibre, two stainless steel microfluidic tubes for the solutions flowing in and out, and a 3D-printed chamber. Finally, all these components were assembled, and all joints were sealed with superglue to ensure that no bubbles were produced in solution samples. 

### 2.3. Data Interpretation

The optical transmission signal was collected by an optical spectrometer (USB4000, Ocean Insights, Orlando, FL, USA) with a resolution of 0.1 nm. The data were manually recorded before and after every experiment step and then analysed at the end of the experiment. SpectraSuite exported the automatically recorded data every 5 s during the experiments, which communicated with Matlab (Mathworks, Natick, MA, USA) to immediately visualize the real-time responses of the sensors during an experiment.

### 2.4. Sensitivity Test

The flow cell was alternately injected with water, 5% NaCl, 10% NaCl, and 20% NaCl solutions to investigate the transmission signal change when the refractive index of the NaCl solution increased.

### 2.5. Protein A Immobilisation Using Spacer Arm and Aero-Length Crosslinker

The gold film was first functionalised with 20 mM of cystamine in Mili-Q water for 1 h. The sensing surface and the flow cell were rinsed with 100 µL of Mili-Q water, and a phosphate-buffered saline (PBS) buffer (pH = 7.4) was used as flow medium for the following steps. A mixture solution of 0.005 M SM(PEG)2 (Sigma Aldrich #22102, St. Loui, MO, USA) and 0.01 M EDTA in PBS (pH = 7.4) was pumped into the flow cell to attach the SM(PEG)_2_ spacer arm to the amine functional group of cystamine via NHS ester reaction. The flow cell was rinsed with 100 µL of PBS buffer to remove SM(PEG)_2_ and EDTA residues upon completion of the reaction. The protein A solution (Sigma Aldrich, St. Loui, MO, USA)) at 1 mg/mL that was reduced in 0.02 M tris(2-carboxyethyl) phosphine (TCEP) (Sigma-Aldrich, St. Loui, MO, USA)) in PBS (pH = 7.4) for 2 h was injected into the flow cell to attach protein A to the SM(PEG)_2_ molecules. After 70 min of protein A immobilisation, the excess of protein A was rinsed with 100 µL of PBS buffer. Unspecific binding sites on the gold film after protein A immobilisation were blocked with 1% of bovine serum albumin (BSA) in PBS buffer (pH = 7.4) for 30 min. 

In another experiment, protein A was immobilised on the sensing surface using a zero-length crosslinker to study the contribution of the spacer arm to the biosensor’s detecting sensitivity. Protein A was immobilised to a 3-Mercaptopropionic acid (3-MPA) self-assembled monolayer (SAM) on the gold film via carbodiimide crosslinking reaction. The gold sensing surface was first functionalized with 20 mM of 3-MPA in Mili-Q water for 1 h. A total of 200 µL of a mixture solution of 0.2 M 1-ethyl-3-(3-dimethyl aminopropyl) carbodiimide hydrochloride (EDC) and 0.05 M N-hydroxysulfosuccinimide (Sulfo-NHS) was injected into the flow cell to create a SAM monolayer. The surface was quickly rinsed with PBS buffer for 1 min. A total of 1.5 mL of 1 mg/mL of protein A was pumped through the surface for 70 min to allow for the immobilisation of protein A on the SAM on the surface. The immobilising reaction was quenched using 300 µL of 0.1 M ethanolamine. Unspecific binding sites were blocked with 1% of bovine serum albumin (BSA) in PBS buffer (pH = 7.4) for 30 min.

### 2.6. Monoclonal Antibody Detection

The anti-β-amyloid monoclonal antibody (Mab) produced in mouse (Sigma Aldrich A8354, St. Loui, MO, USA)) (Molecular Weight = 110 kDa) was used to evaluate this sensing platform. The Mab solutions were prepared at a concentration ranging from 10^−5^ mg/mL to 10^−1^ mg/mL. A total of 200 µL of the Mab solution was pumped into the flow cell at an ascending concentration. After each measurement, the gold film was rinsed with 100 µL of PBS buffer. The flow rate was kept at 20 µL/min throughout the experiment. 

For the experiments investigating monoclonal antibody detection with and without a spacer arm, the antibody samples were injected into the flow cell in the order of ascending concentrations ranging from 10^−5^ mg/mL to 10^−2^ mg/mL. Each sample was injected for 10 min to measure the wavelength shift. The surface was rinsed with PBS buffer for 5 min to remove any excess antibody on the sensing surface and become ready for the following sample.

### 2.7. Protein A Regeneration

The regeneration of Protein A was carried out in two attempts, and the same sensing gold film was used in both attempts. In the first regeneration attempt, after 200 µL of antibody solution was applied at a 0.01 mg/mL concentration for measurements, the flow cell was rinsed with 100 µL of PBS buffer and 50 µL of 0.1 M glycine hydrochloride (pH = 2.8) as a stripping solution. The flow cell was equilibrated with 100 µL of PBS buffer. The above process was repeated five times for the first regeneration attempt. After the first regeneration attempt, the immobilised protein A on the sensor surface was stored at 4 °C in PBS buffer with 0.01 M EDTA for one week before the second regeneration attempt. In the second regeneration attempt, the sensing gold film was treated with 6 M guanidine hydrochloride in Mili-Q water for 5 min to remove residual proteins or antibodies on the sensing surface. Five regenerating tests were carried out, similarly to the first attempt. 

## 3. Results

### 3.1. Fabrication of Fibre-Optic EOT Biosensor-Incorporated Mab Detection Device

One of the most remarkable features of the EOT biosensors compared to the traditional SPR biosensors is that it offers a more straightforward optical setup, facilitating miniaturisation of the sensor platform. To further improve the portability of fibre-optic EOT biosensors and address the engineering issues in the previous design by Jia and Yang [38], modifications were made to the biosensor design on the original prototype. In the original prototype, polydimethylsiloxane (PDMS) was used to fabricate a flow cell; optical fibres and microfluidic tubes were attached to the flow cell by press fits. There were three engineering issues associated with this biosensor prototype. First, the flow cell made from PDMS was quite fragile and not applicable to long-term usage. Second, the optical fibres used in the prototype had sleeves and connectors. These optical fibres could not provide a completely confined and airtight seal for the flow cell due to gaps between the sleeves and connectors of optical fibres. Consequently, there was a risk of air bubbles in the flow cell during measurements, and air bubbles would interfere with the detection of signals. Third, the optical fibre for the prototype had multiple structural components, but these components were not essential for the biosensor. These structural components accounted for an extra cost, and led to a heavy sensor and complex instrument.

**Figure 1:**
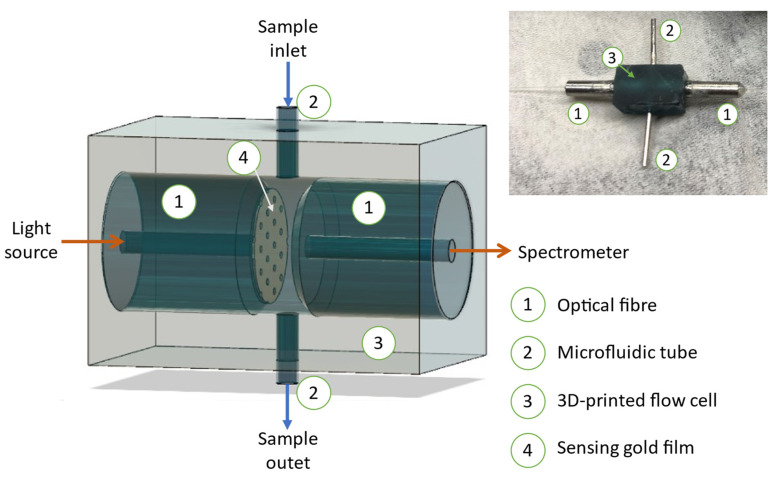
3D sketch simulates the flow cell after assembling with optical fibres and microfluidic tubes, and a photo of an actual flow cell after assembly.

Modifications were made to address the above issues and improve the portability and overall performance of the fibre-optic EOT biosensor for Mab measurements. Redesigning flow cells and selecting suitable materials significantly improved the airtightness and sturdiness of the fibre-optic EOT biosensor. Figure 1 illustrates the 3D simulation of the flow cell and the actual flow cell fabricated by a 3D printer. The chamber of the flow cell. was designed using 3D CAD sketching software to ensure it could be tightly connected to the optical fibres and microfluidic tubes. It was fabricated by a 3D printer using a photopolymer resin, since this material is more durable than PDMS and can be reused multiple times. Second, only the core of optical fibres and a stainless-steel ferrule were used to fabricate the fibre-optic EOT biosensor to reduce the cost and weight of the biosensor. They were assembled with epoxy glue to prevent air-leaking gaps between the optical fibre and the stainless-steel ferrule. Finally, all individual components, including the flow cell’s chamber, two optical fibres, and two microfluidic tubes, were assembled by a superglue to complete the flow cell.

The weight of the flow cell is approximately 5 g with a dimension of 6 × 6 × 10 mm, which offers good portability and great potential for on-line or point-of-care applications. 

### 3.2. Sensitivity of the Fibre-Optic EOT Biosensor

**Figure 2:**
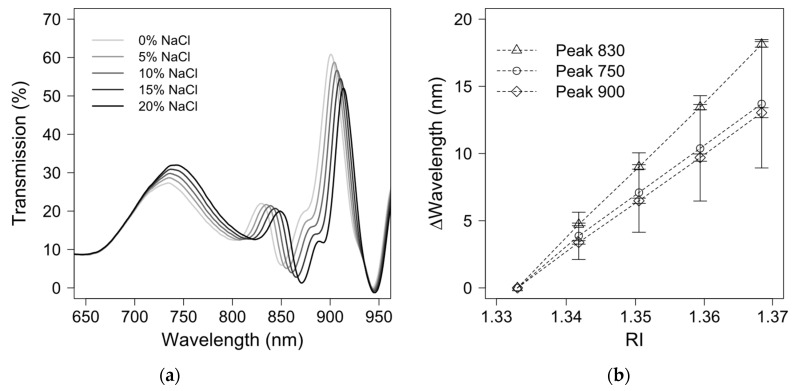
(**a**) Wavelength shifts in optical transmission at different NaCl concentrations for sensitivity tests; (**b**) Average wavelength shifts in optical transmission peaks at different NaCl concentrations from three sensing probes.

Three sensing probes were prepared for Mab detections. Before applying these probes to Mab detections, their sensitivity was determined. NaCl solutions at 0%, 5%, 10%, 15%, and 20%, corresponding to a refractive index (RI) of 1.3330, 1.3418, 1.3505, 1.3594, and 1.3684, respectively, were pumped into the flow cell and the optical transmission spectra were recorded (Figure 2a). A redshift was observed in the optical transmission wavelengths when the NaCl concentration increased. A higher NaCl concentration at a higher RI is correlated with a larger redshift in the optical transmission spectra.

The wavelength shifts in the optical transmission peaks of three probes were evaluated at different reflective indexes, as shown in the scatter graphs of Figure 2b. Overall, the wavelength shift has an excellent linear correlation with the refractive index. Prominent peaks were chosen at 750 nm, 830 nm and 900 nm, because these were present in all optical transmission spectra, are highly distinguishable from other peaks and shoulders, and have decent symmetry. These prominent peaks also allow for feasible curve-fitting for data analysis. In Figure 2b, the peak at 830 nm has the most significant redshifts in the optical transmission spectra, with very consistent sensitivity among the three probes (509 ± 5 nm/RIU) in comparison with the peaks at 750 nm (383 ± 130 nm/RIU) and 900 nm (366 ± 10 nm/RIU). The sensitivity of the peak at 900 nm has a good consistency for the three probes; however, the peak at 900 nm is less sensitive than the peak at 830 nm at different RI values. Although the redshifts of the peak at 750 nm are distinct as the RI value increases, they are less consistent than those at 900 and 830 nm, which might be due to the spectral error resulting from the imperfection and roughness of the gold film. According to finite-difference time-domain (FDTD) simulations, the peak at 750 nm can be attributed to the local surface plasmon resonance inside the nanoholes when the peak at 830 nm and 900 nm may originate from the top edge of the nanohole and the bottom surface of the gold film. The simulation result agrees well with the sensitivity test result because peaks at 750 nm, 830 nm, and 900 nm shift significantly to the RI change of the NaCl solution. Details of the simulated result are included in the Supplementary document. ANOVA on the sensitivity of peaks at 750 nm, 830 nm, and 900 nm across three sensing probes reveals that the mean values for the sensitivity from three sensing probes at three peaks are not significantly different (*p* = 0.12). Additionally, t-test analysis suggested that the sensitivity of the peak at 830 nm is significantly different from that at 900 nm (*p* = 0.002), while there is no statistical difference for the sensitivity between 750 nm and 830 nm (*p* = 0.123). Since there is a large deviation in the sensitivity of the peak at 750 nm across three sensing probes, the peak at 830 nm is chosen for data analysis in the final analysis due to its excellent sensitivity and consistency.

The minimal variations in the sensitivity of the peak at 830 nm among three probes indicate the excellent reproducibility of the template transfer procedure in the probe preparation process. It is critical to ensure that the template is placed vertically against the optical fibre during the template transfer process. It was reported that the sensitivity of an EOT biosensor could be tuned by changing the light incident angle [5], which indicates that, despite their sharing the same nanohole array, with the same pattern and the same silicon template, the sensitivity could vary due to the different angles between the template and the optical fibre during the template transfer process. We used a microscope camera to monitor the template transfer process and ensure that the template was vertical to the optical fibre.

Minor variations in the sensitivity in Figure 2b could be further mitigated by a well-controlled template transfer process using robotic arms. It is impossible to examine the consistency of the sensitivity in previous studies, especially those based on multi-channel microfluidic chips, because these experimental results were presented from a single transfer [28,31,32,36,37]. Maintaining consistency in the sensitivity during the template transfer is vital because it dramatically impacts the feasibility of commercialising fibre-optic EOT biosensors for application in Mab titer measurements. Our fabrication process is demonstrated to have excellent reproducibility in terms of sensitivity from three template transfers when the process is well-controlled.

### 3.3. Monoclonal Antibody Detection

The fibre-optic SPR-EOT biosensor was tested to quantify Mab concentrations in a real-time and label-free manner. The experiments were designed to demonstrate that (1) the measured antibody concentration results could be reproducible; (2) the protein A immobilisation process could be simplified without spacer arms; (3) the sensor could be reused after regeneration.

**Figure 3:**
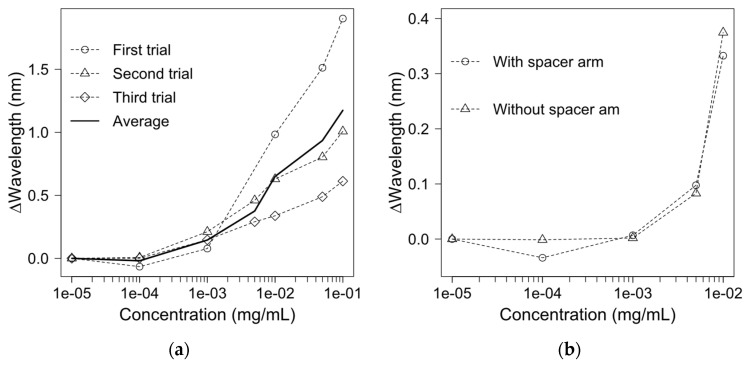
(**a**) Antibody detection using protein A-based fibre-optic SPR-EOT biosensors in triplicate with spacer arm; an average of three replicates is shown as a solid line; (**b**) Impact of spacer arms for immobilising protein A on the detected results.

To detect and quantify the Mab concentrations, protein A was immobilised onto the gold film, since it has a high affinity for the antibody. A murine monoclonal anti-β-amyloid antibody IgGa2 was chosen as a target protein because of its strong interaction with protein A [39]. The antibody concentration in the experiment tests ranged from 10^−5^ mg/mL to 0.1 mg/mL. The experiment was carried out in triplicate to obtain an average standard curve, as shown in Figure 3a.

The signal starts to noticeably shift when the Mab concentration is beyond 10^−3^ mg/mL. As the concentration increases, the wavelength is significantly shifted. From the three trials, a similar limit of detection (LOD) is found for the Mab. The average LOD is calculated as 0.44 µg/mL. Large deviations are observed among the three tests at a high antibody concentration. The deviations could be ascribed to the manual fabrication process for the optic EOT-based biosensor. It is worth mentioning that these measurements were carried out for Mab samples prepared from different batches. An automatic fabrication process for the biosensor for the same batch Mab samples could significantly reduce the variations at a high protein concentration.

### 3.4. Monoclonal Antibody Detection Using a Zero-Length Crosslinker

The effect of the spacer arm between immobilised protein A and the gold film on Mab detection was investigated, and the results are depicted in Figure 3b Spacer arms were demonstrated to improve the detection sensitivity, reduce non-specific binding and assist in oriented protein immobilisation [40]. However, the employment of a spacer arm in an immobilising process contributes to an additional cost for the materials and a long preparation time. Hence, we compared the detection sensitivity of Mab titers with and without a spacer arm. Protein A was immobilised either with a spacer arm SM(PEG)2 or directly onto the gold sensing surface using zero-length carbodiimide crosslinking without this spacer arm. The results suggest that the spacer arm does not significantly boost the detecting sensitivity, since the shift is 0.333 nm with a spacer arm at an antibody concentration of 0.01 mg/mL, and 0.375 nm without a spacer arm (as Figure 3b) Our results do not align with the conclusion from a previous study, in which SMPEG, when used for oriented protein immobilization, could lower the LOD and significantly reduce non-specific binding [40]. The unexpected difference in these experimental results could be due to the difference in antibody–antigen and protein A–IgG binding interactions. The orientation of an antibody is crucial for antibody–antigen interactions because antigens can only bind to the fragment antigen-binding (Fab) region. When the Fab region is affected by the immobilisation process, the detection ability of the biosensor could be severely impaired. For Protein A, each protein A could accommodate up to two IgGs, because the binding ratio between protein A and IgG is 1:2 [41]. When one binding site of protein A is hidden during the immobilisation process, IgG molecules could bind to another binding site of protein A for detection by the sensor. In our case, the spacer arm could help to enhance the flexibility of the immobilised protein A, but could not oriente protein A molecules during immobilisation. However, due to the considerable size difference between protein A (42 kDa) and IgG (110 kDa) [41], the hindrance effect could occur when IgG binds to a layer of protein A that is immobilised onto a 2D surface; thus, the contribution of the spacer arm in improving the protein flexibility could become negligible.

### 3.5. Protein A Regeneration

**Figure 4:**
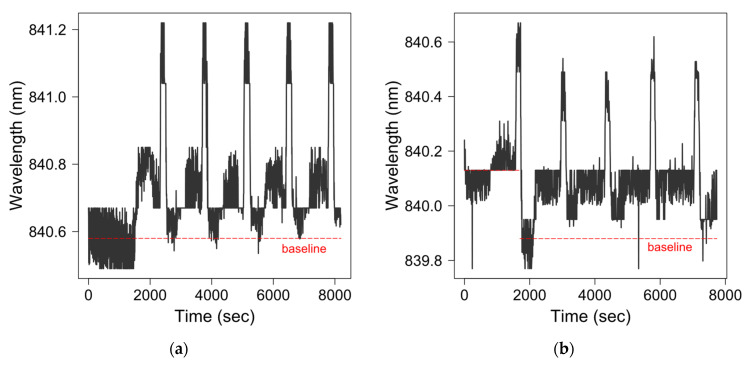
Sensor grams of two protein A regeneration attempts. (**a**) First attempt with five runs of protein A regeneration: (**b**) Second attempt on seven days after the first one with five protein A regenerations.

Finally, we investigated the feasibility of using the fibre-optic SPR-EOT biosensor as a PAT tool to continuously monitor the Mab titer in a bioreactor for a fed-batch operation. Two different regeneration attempts were conducted for protein A, using the same sensing probe, immobilised with protein A. In the first regeneration attempt, the sensor was tested with five sample injections (0.01 mg/mL antibody). After each measurement, 0.1 M glycine hydrochloride (pH = 2.8) was applied to strip IgG molecules, and protein A was regenerated after equilibration with the PBS buffer. After five regenerations in the first attempt, the sensor was stored for a week. The sensing surface was then cleaned with 6 M guanidine hydrochloride in Milli Q water for 5 min to dissolve the residue of the sample Mab before a new sample was charged [42]. Another five samples were processed in a similar procedure to the first attempt. The sensorgrams of both trials are shown in Figure 4. Two graphs start from a baseline, where the medium is PBS buffer (pH = 7.4), and no Mab is bound to the immobilised protein A. The signal starts to increase after the injection of 0.01 mg/mL Mab (pH = 7.4) in 10 min. The signal change is maintained even after the sensing surface is rinsed with a PBS buffer (Ph = 7.4) for 5 min. The sensing surface is then regenerated by a quick flush with 0.1 M glycine hydrochloride (pH = 2.8), and a sharp and defined peak appears in the sensorgrams. After regeneration, the flow cell is rinsed with PBS buffer (pH = 7.4), and the transmission signal returns to the baseline before the next regeneration experiment. According to the sensorgram of the first attempt (Figure 4a), the signal does not return to the baseline after the first regeneration; however, the wavelength shifts due to Mab injection after each regeneration are very consistent. An average wavelength shift of 0.126 ± 0.003 nm at an antibody concentration of 0.01 mg/mL is obtained for five regenerations after stripping the sensing surface with a strong acid. The signal shift cannot return to the initial baseline after the first regeneration, indicating that there are sample residues on the sensing gold film. Although 0.1 M glycine hydrochloride (pH = 2.8) is often used in a protein A column to remove Mab residues before equilibration, Mab samples may be more strongly bound to protein A on the 2D sensing platform than in a protein A column.

In the second regeneration attempt (Figure 4b), guanidine hydrochloride was used to strip the Mab residues after Mab was injected into the flow cell. According to the sensorgram, the signal drops below the initial baseline after the first regeneration in the second attempt. It is possible that the combination of guanidine hydrochloride then 0.1 M glycine hydrochloride (pH = 2.8) might have stripped more residual Mab than guanidine hydrochloride alone. After the first regeneration attempt, the average signal shift in antibody detection increases to 0.167 ± 0.002 nm, indicating that the sensing surface has fewer Mab residues, while protein A still maintains its bioactivity. The sensing surface is coated with protein A, a very robust protein, as a detecting ligand, so that the signal could be specific to Mab molecules; the sensing surface could be reused many times by regenerating the protein A surface after each use. Therefore, the sensor could have an excellent potential for the continuous monitoring of Mab samples in a bioreactor.

## 4. Discussion

In recent years, optical biosensors have received great attention due to their advantages over other types of sensors. The most distinctive advantages include their excellent sensitivity and specificity, compact size, and label-free detection, making optical biosensors a perfect candidate for point-of-care or at/in/on-line monitoring applications [43]. They have been extensively studied for their various biological applications such as medical diagnosis, pharmaceutical/food process control, or the detection of chemical residues in the environment/food/water [44]. Despite an increasing number of studies on optical biosensors, few of them have been applied to routine and commercial use [44,45]. Factors that hinder the commercialisation of these optical sensors include the poor stability of the immobilised protein, high cost of the fabrication of sensors, low throughput without miniaturised optical biosensors, and insufficient validity [45]. In this study, the regeneration experiment demonstrated that the immobilised protein A could become stable, with similar bioactivity, after one week at 4 °C. Protein A can be immobilised via a zero-length crosslinker to simplify the fabrication procedure.

Additionally, the cost of fabricating a sensing flow cell is around 15 Australian dollars at a laboratory scale, and the flow cell parts are sterilisable and reusable. Compared to other PATs for measuring Mab titers, the cost of fabricating fibre-optic SPR-EOT biosensors is low; the production cost could be even lower at a larger manufacturing scale. The flow cell is compact and portable, with great potential for implementation in a GMP environment. Throughput could be improved by implementing parallel flow cells into the biosensor.

Ultimately, the proof-of-concept using of the fibre-optic SPR-EOT biosensor to quantify Mab samples as an at-line/on-line PAT tool has been demonstrated. Our antibody detection result is comparable to the results from a previous study [24]. A 96-well EOT biosensor had a sensitivity of 900 nm/RIU and the LOD of 5 nM for IgG detection (equivalent to 0.75 µg/mL, assuming that the MW of IgG is 150 kDa). The overall peak shift was 1.8 nm at the IgG concentration ranging from 1.5 × 10^−5^ to 1.5 × 10^−1^ mg/mL. This overall shift is greater than ours, which is less than 1.5 nm over the concentration range from 10^−5^ to 10^−1^ mg/mL. The difference in overall shifts is due to the sensitivity of the two different systems (900 nm/RIU for 96-well EOT biosensor vs. 509 nm/RIU for our biosensors).

Considering the increasing development of therapeutic proteins toward a continuous and automated operation, fibre-optic SPR-EOT biosensors could be applied to the real-time monitoring of Mab titers in an automated feedback control system [1]. Comparing the methods used to measure Mab samples using HPLC or derive the Mab titer by monitoring the biomass/metabolites concentration, the fire-optic SPR-EOT biosensor is cheap, portable, and rapid in the detection of Mab molecules in the sample, and it is also easy to operate and implement. HPLC with automated sampling is the most accurate chromatographic method for at-line detection of Mab titers; however, it is resource-intensive, laborious, and requires a considerable amount of space [1]. On the other hand, Raman spectrometry is the most attractive in-line optical method for quantifying titers [1]. However, the Raman signal is often very weak. The interpretation of the Raman spectra for a Mab sample includes many impurities, including host cell proteins, DNA/RNA, metabolites and Mab variants, which can be very challenging [42]. Our fibre-optic SPR-EOT biosensor is a potential PAT for the continuous monitoring of Mab samples in a bioreactor. It can rapidly quantify the Mab concentration at-line, similar to other optical methods (e.g., pH, O2, and temperature sensors), while providing a similar accuracy to HPLC. 

Despite efforts into developing novel technologies for real-time acquisition of Mab titer data in a bioreactor, very few of these technologies are applied for routine use in the biopharmaceutic industry because of strict regulations. Robustness, reproducibility and accuracy of new PATs have to be addressed to meet the requirements from regulatory agencies [2]; therefore, it is crucial to comply with the guidelines during the device development to optimise the opportunities of commercialisation. Our fibre-optic SPR-EOT biosensor is made from sterilisable materials with a compact size, so it could be easy for implementation, maintenance, and replacement in a GMP environment as an at-line tool to measure Mab titers. Besides, it can be readily adaptable to previously developed automatic sampling systems to make the measurement process completely automated.

## 5. Conclusions

We have demonstrated a portable and sensitive fibre-optic SPR-EOT biosensor for the rapid detection of biomolecules, especially for monitoring the product concentration during bioprocesses. After improving the flow cell design, the biosensor can be operated with minimal interference of air bubbles and other external factors. A consistent sensitivity between template transfers is achieved with precise control during the fabrication procedure for three sensing probes. A similar detection limit from three antibody detection tests confirms the repeatability of this biosensor. The biosensor could detect antibodies at a concentration of as low as 0.44 µg/mL. The redshifts in the optical transmission spectra correlate with the Mab concentration, although significant variations were observed at a higher Mab concentration. Additionally, the spacer arms for immobilising protein A onto the gold film does not improve the detecting sensitivity; therefore, protein A could be immobilised with a zero-length crosslinker to save operating time and material cost. The biosensor could be regenerated for the measurements of Mab concentrations up to ten times. From this proof-of-concept using the fibre-optic SPR-EOT biosensor for the rapid detection of Mabs, it can be concluded that this biosensor is potentially beneficial for at-line measurements of product concentration in the manufacturing process of Mabs, because of its acceptable repeatability, portability, and user-friendliness.

## Data Availability

Data supporting results can be found in the Supplementary document.

## Supplementary Materials

The following are available online at https://www.mdpi.com/article/10.3390/bios11100383/s1. Figure S1: Flow cell’s chamber design with details about dimensions, Scheme S1: Step-by-step diagram of fabricating the gold sensing surface with a periodic nanohole matrix using a nanopatterned silicon template and epoxy glue, Figure S2: Transmission spectra in different NaCl concentrations obtained from experiment and simulation, Figure S3: Electrical field distribution in water of the simulated resonance peaks and simulated sensitivity results, Figure S4: Step-by-step diagram of protein A immobilisation with and without spacer arm, Figure S5: Schematic diagram explaining the procedure of regeneration epxeriment, Figure S6: Detecting results after two regneration trials.
