# Supplementary material for: Fibre-Optic Surface Plasmon Resonance Biosensor for Monoclonal Antibody Titer Quantification
Source: Biosensors (Basel). 2021 Oct 10;11(10):383. doi: 10.3390/bios11100383 (PMC8534111; doi:10.3390/bios11100383)
Supplement: Supplementary file 1 [file biosensors-11-00383-s001.zip › biosensors-1393415-supplementary.pdf]

biosensors

MDPI

Supplementary Materials

Fibre-Optic Surface Plasmon Resonance Biosensor for Monoclonal Antibody Titer Quantification

Thai Thao Ly [1], Yinlan Ruan [2,3,*], Bobo Du [4], Peipei Jia [5] and Hu Zhang [6,*]

1 School of Chemical Engineering and Advanced Materials, University of Adelaide, Adelaide, 5005, Australia; thaithao.ly@adelaide.edu.au[
2 Institute for Photonics and Advanced Sensing, University of Adelaide, Adelaide 5005, Australia
3 School of Electronic Engineering and Automation, Guilin University of Electronic University, Guilin 541004, China.
4 Key Laboratory for Physical Electronics and Devices of the Ministry of Education, School of Electronic Science and Engineering, Xi'an Jiaotong University, Xi'an 710049, China; bobo.du@xjtu.edu.cn
5 Shenzhen Institute for Advanced Study, University of Electronic Science and Technology of China, Shenzhen 518000, China; jiapeipei@uestc.edu.cn
6 Keck Graduate Institute, Claremont, CA 91711, USA; hu_zhang@kgi.edu
* Correspondence: Yinlan.ruan@adelaide.edu.au (Y.R.); hu_zhang@kgi.edu (H.Z.)

S1. Flow Cell Design

Figure S1 depicts the 3D design of a new flow cell chamber for the fibre-optic SPR-EOT biosensor. The inserts are designed with an appropriate clearance to accommodate optical fibres of Ø = 2.5 mm and microfluidic tubes of Ø = 0.8 mm. All these inserts are designed with fillets to assist in flow cell assembly. The distance between two optical fibres is controlled to be 100 μm or less using a microscope camera.

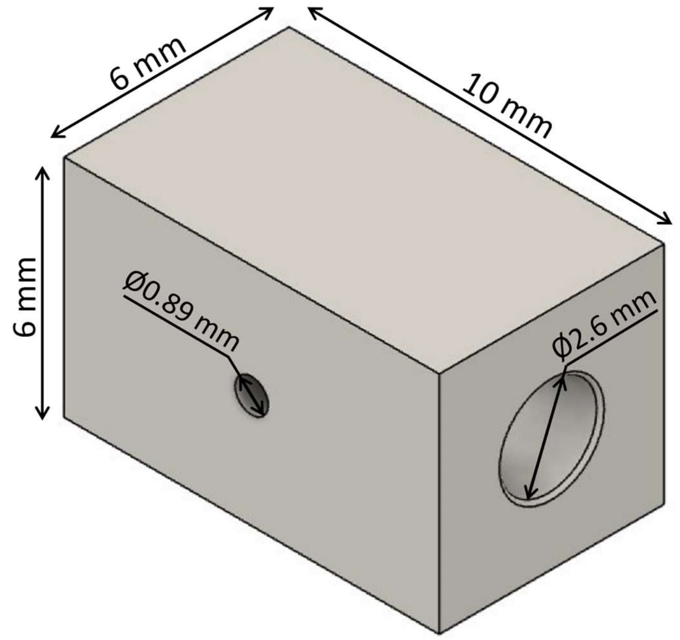

Figure S1. Flow cell's chamber design with details about dimensions.

Template Transfer Procedure

The fabrication procedure is depicted in Error! Reference source not found.**,** with the details of each step in the caption. The procedure is divided into three major tasks: cleaning the template and depositing a gold film; cleaning the optical fibre; template transfer using epoxy glue. Large variations may occur during the template transfer process in

Step (E) in the fabrication of different sensors; therefore, a microscope camera is used to ensure the optical fibre is vertical to the template to mitigate variations.

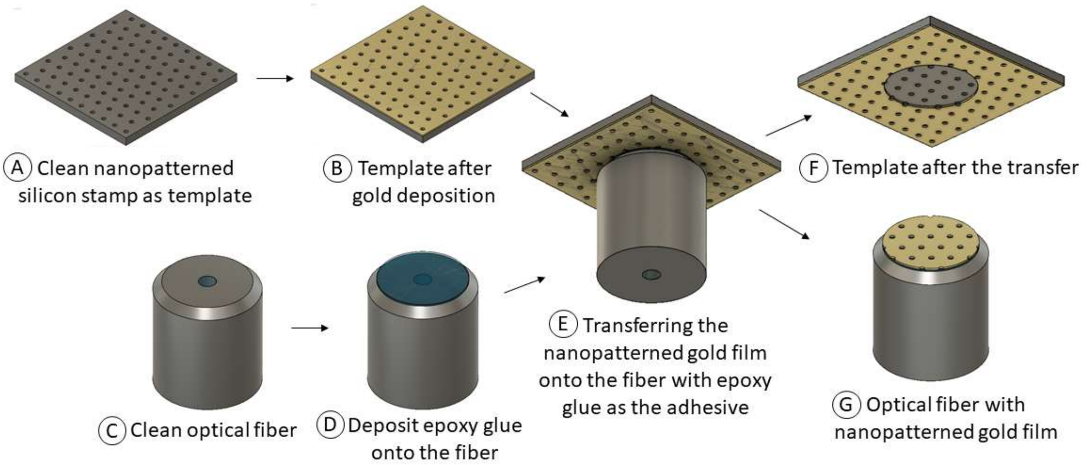

Scheme 1. Step-by-step diagram of fabricating the gold-sensing surface with a periodic nanohole matrix using a nanopatterned silicon template and an epoxy glue. (**A**) Thoroughly cleaning the nanopatterned silicon template; (**B**) Coating 100 nm of gold nanoparticles onto the silicon template; (**C**) Cleaning an optical fibre; (**D**) Depositing an epoxy glue layer onto the fibre; (**E**) Attaching the optical fibre with an epoxy glue to the nanopatterned gold film; (**F** + **G**) Transferring the nanopatterned gold film onto the tip of optical fibre.

S2. Simulation Results

Figure S2 shows the transmission spectra in different NaCL concentrations, obtained from the experiment and simulation. The peak at around 500 nm is characteristic of gold and present in both experimental and simulated spectra. The measured peak at 750 nm can be related to the simulated peak at 683 nm. This peak can be attributed to the coupling between the local surface plasmon resonance at the top and bottom corners of the nanoholes. The measured peaks at 830 nm and 900 nm can be related to the simulated peak at 882 nm, which originates from the water/Au mode. The multiple peaks in the experiments could result from the imperfection and roughness of the gold film. The measured peak at 980 nm can be related to the simulated peak at 991 nm, which originates from the epoxy/Au mode.

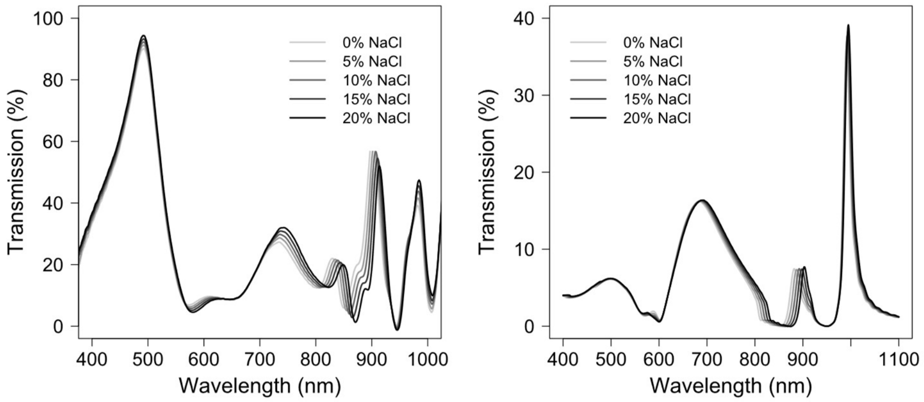

Figure S2. Transmission spectra in different NaCl concentrations obtained from (**a**) experiment and (**b**) simulation.

The resonance peaks can be demonstrated and confirmed by the corresponding electric field distributions, as shown in **Figure S3**. In **Figure S3**, the refractive index sensing in experiments and simulations is summarized. For water/Au mode, the sensitivity of peak 880 nm from the simulation is comparable to the sensitivity of peak 830 nm in

experiments (509 ± 5 nm/RIU and 593 nm/RIU, respectively). For the epoxy/Au mode, the measured peak at 980 nm has an average sensitivity of 141 ± 70 nm/RIU, which is approximately the simulated sensitivity of 107 nm/RIU of peak 991 nm. However, the peaks at 683 nm in simulations and 750 nm in experiments show a large divergence (193 nm/RIU and 383 ± 130 nm/RIU, respectively), which might be due to the spectral error resulting from the from the imperfection and roughness of the gold film.

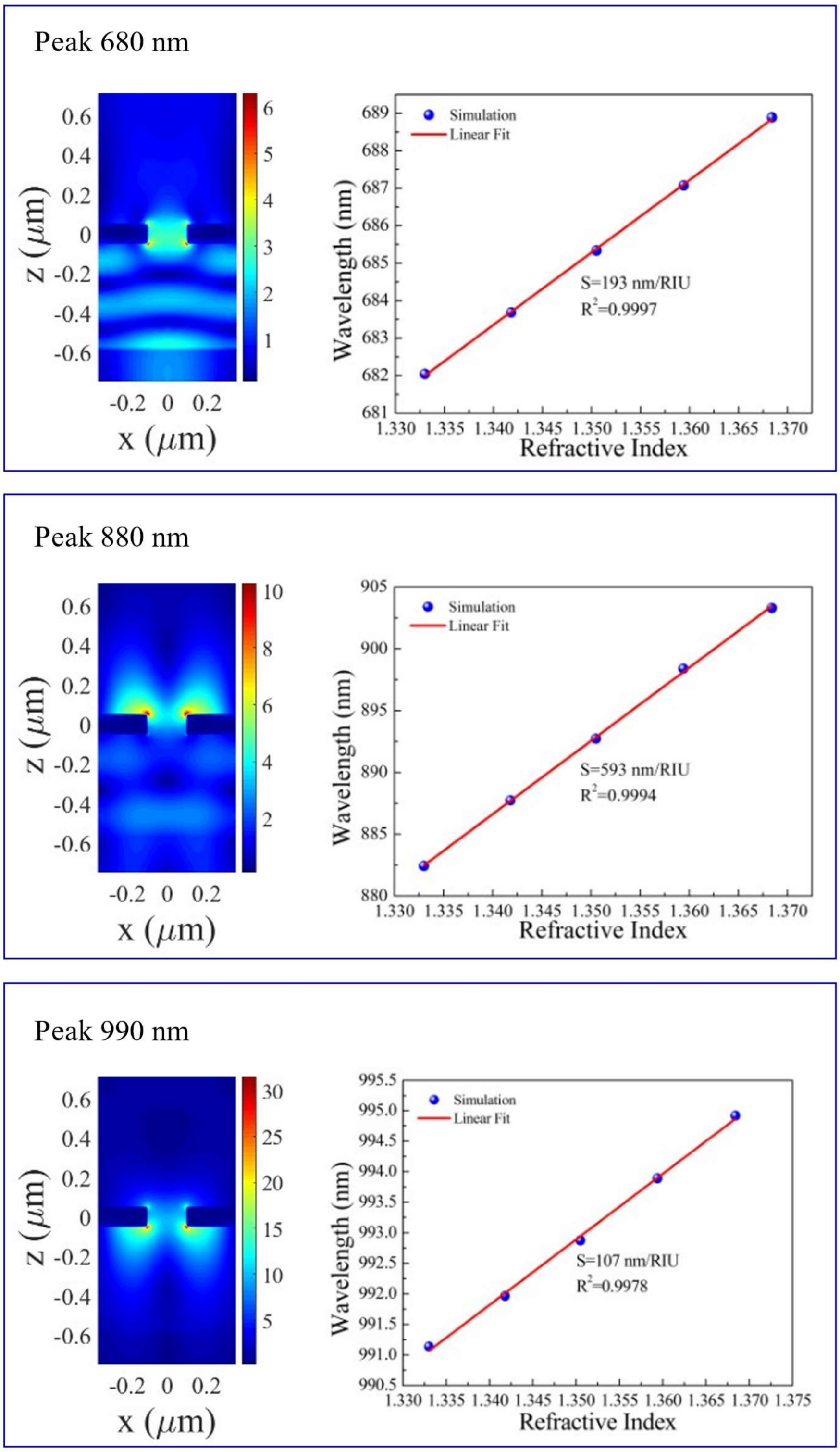

Figure S3. Electrical field distribution of the simulated resonance peaks in water and simulated sensitivity results.

S3. Protein A Immobilisation

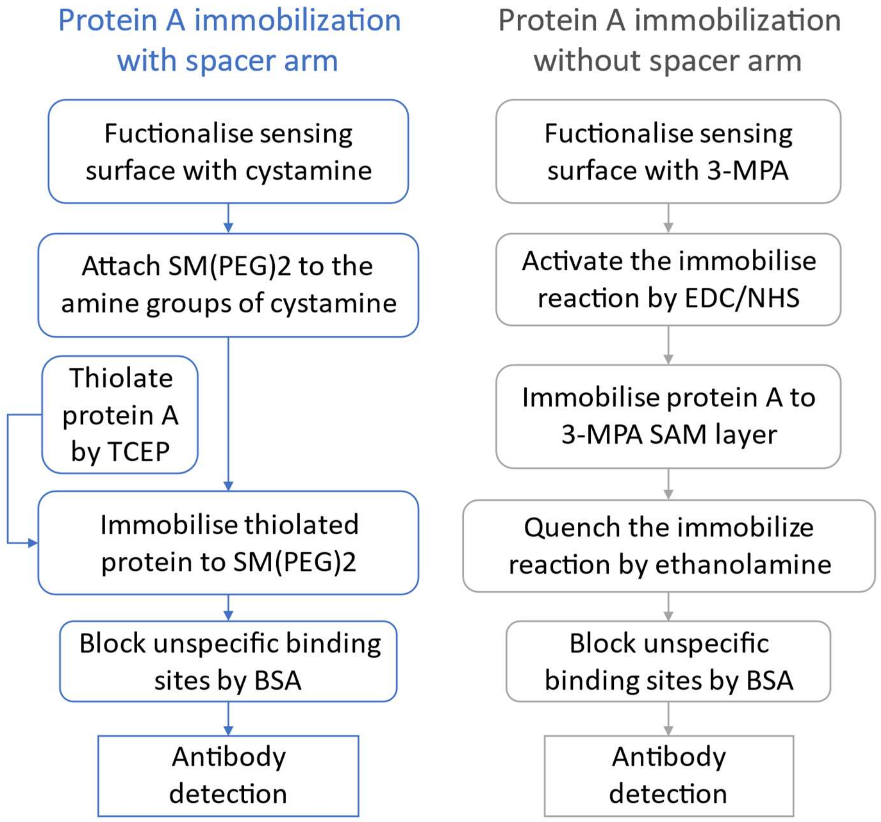

Figure S4. Step-by-step diagram of protein A immobilisation with and without spacer arm.

Figure S4 outlines the steps in protein immobilisation with and without a spacer arm. The diagram is included to provide a better visualisation of the complicated procedure.

S4. Protein A Regeneration

A diagram explaining the two attempts at a regeneration experiment is provided in Figure S5.

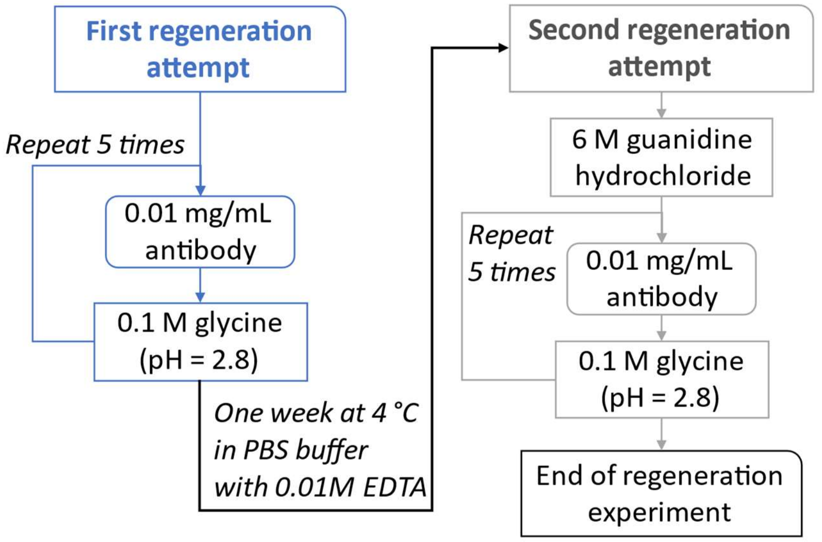

Figure S5. Schematic diagram explaining the regeneration experiment procedure.

The detected results of the two attempts at the regeneration experiment are shown in Figure S6. The detected wavelength shifts are pretty consistent for each trial. The peak shift obtained from the sixth regeneration, which is the first regeneration of the second trial, is not included in the figure because it was not consistent with other values obtained in both attempts. The sixth regeneration was conducted after treating the sensing surface with 6 M guanidine hydrochloride and returned a peak shift of 0.095 nm after detecting 0.01 mg/mL Mab. This peak shift result is remarkably lower than all the peak shifts acquired during the regeneration experiment. It may be that there were still denatured Mab residues on the sensing surface after treatment with 6 M guanidine hydrochloride, which hindered the binding of the Mab, and all these residual Mabs were stripped by 0.1 M glycine hydrochloride (pH = 2.8) during the sixth regeneration. After that, the peak shift dropped below the initial baseline of the second trial and the detected signals were consistent from the seventh regeneration onward (as shown in Figure S6).

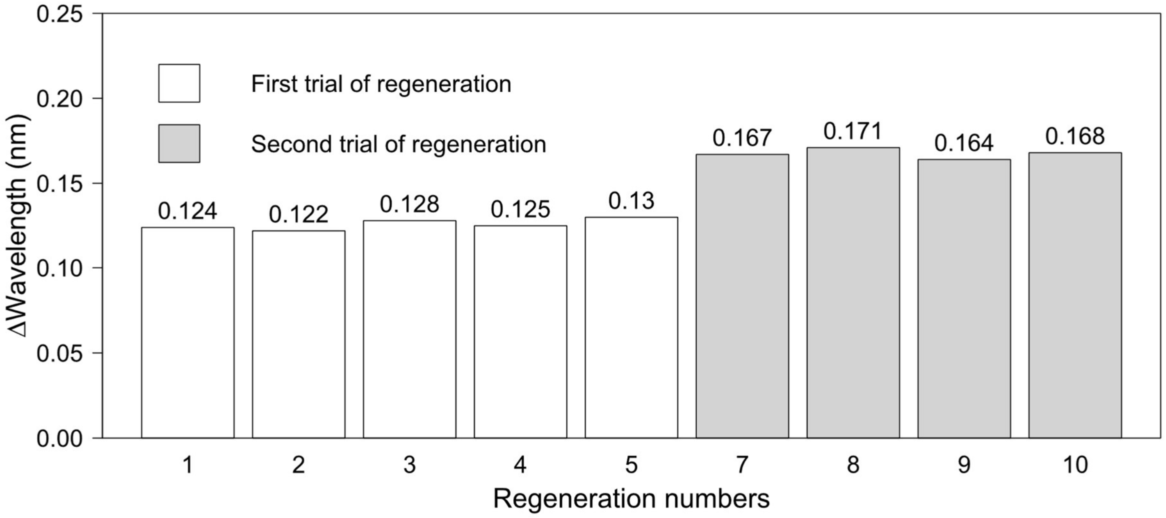

Figure S6. Detected results after two regeneration trials. The sensing surface with protein A was cleaned with 6 M guanidine hydrochloride before the second trial.
